# Multiband Optical Physical Unclonable Functions Based on Mixed‐Phase Multilevel Emissive Perovskites

**DOI:** 10.1002/advs.78505

**Published:** 2026-11-10

**Authors:** Seungbeom Park, Jiyoun Kim, Jiyoung Boo, Giho Kim, Jinsik Yoon, Junghyun Bae, Sangeun Cho, Wook Park, Dong Hyuk Park, Healin Im

**Affiliations:** ^1^ Department of Applied Physics Kyung Hee University Yongin Republic of Korea; ^2^ Department of Chemical Engineering Inha University Incheon Republic of Korea; ^3^ Program in Biomedical Science and Engineering Inha University Incheon Republic of Korea; ^4^ Institute for Advanced Fiber Convergence Materials Kyungpook National University Daegu Republic of Korea; ^5^ Department of Chemical Engineering Kyung Hee University Yongin Republic of Korea; ^6^ Department of Electronic Engineering College of Engineering & Information Kyung Hee University Yongin Republic of Korea; ^7^ Division of System Semiconductor Dongguk University Seoul Republic of Korea; ^8^ Institute for Wearable Convergence Electronics Department of Electronics and Information Convergence Engineering Kyung Hee University Yongin Republic of Korea; ^9^ School of Electronic Engineering College of Electronic & Information Kyung Hee University Yongin Republic of Korea

**Keywords:** multicolor emission, perovskite, physical unclonable functions

## Abstract

Physical unclonable functions (PUFs) are hardware‐based security primitives that exploit intrinsic physical randomness in materials or devices to prevent counterfeiting and enhance authentication across diverse applications. Among various sources of randomness, the optical signatures of luminescent materials represent a particularly robust and easily distinguishable feature. However, many optical PUF systems rely on environmentally hazardous materials or energy‐intensive fabrication processes while remaining vulnerable to counterfeiting due to deterministic and spectrally fixed emission characteristics. Here, we demonstrate sustainable multiband optical PUFs by forming heterostructures between lead‐free phase‐mixed perovskites and cellulose fibers via a water‐assisted drop‐casting process. Cesium manganese bromide perovskites provide an environmentally benign composition while exhibiting excitation‐dependent multilevel photophysical responses arising from mixed‐phase interactions. By leveraging these excitation‐dependent multilevel emission characteristics, we construct strong PUFs that significantly expand the challenge and response pairs from a single device through controlled optical feature extraction of the perovskites and the spatial randomness of cellulose fibers. Moreover, the multiband optical PUF platform is demonstrated for image encryption/decryption as well as currency authentication. These results establish an environmentally responsible optical PUF platform that combines sustainable materials and fabrication processes with distinctive photophysical characteristics for next‐generation hardware security technologies.

## Graphical abstract

In this paper, sustainable multiband optical PUFs were developed by constructing heterostructures between lead‐free phase‐mixed perovskites and cellulose fibers via a water‐assisted drop‐casting process. Cesium manganese bromide perovskites provide an environmentally benign composition while exhibiting excitation‐dependent multilevel photophysical responses arising from mixed‐phase interactions.

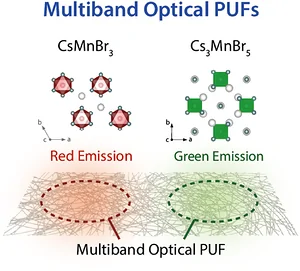

## 1 Introduction

Secure authentication has become a critical requirement in a hyperconnected world supply‐chain logistics, distributed sensing, and the artificial internet of things. With the rapid emergence of machine learning, conventional cryptographic systems, which particularly rely on algorithm‐based random number generation, are increasingly vulnerable due to their deterministic nature and susceptibility to advanced computational attacks. Physical unclonable functions (PUFs) [1, 2] have emerged as a promising hardware‐based alternative, exploiting inherent and irreproducible physical variations introduced during material synthesis or device fabrication. Similar to biometric identifiers, such as fingerprints or iris patterns, PUFs derive security from physically encoded randomness that is fundamentally resistant to duplication, prediction, and emulation. These variations generate a large number of unique challenge‐response pairs (CRPs), forming the basis of robust and tamper‐resistant authentication systems.

A variety of disordered physical characteristics [3] have been explored as entropy sources for random number generation. Among them, optical PUFs have attracted attention owing to their inherent spatial and spectral randomness arising from micrometer‐scale patterns formed under controlled fabrication conditions [4, 5]. Such systems offer high complexity and entropy through precise molecular engineering, such as structural design [6, 7, 8] and stoichiometric control [9, 10, 11] as well as environmental factors including templating effects and interfacial interactions within heterostructures [10, 12]. Most importantly, by expanding the dimensionality of the input space during data collection, strong PUF architectures can be readily realized in optical cryptographic platforms. Although the macroscopic optical patterns appear static, their underlying spectral responses encode hidden randomness that can be selectively accessed through external stimuli, such as excitation wavelength or interfacial coupling with adjacent materials [12, 13]. This approach effectively addresses the intrinsic vulnerability of conventional pattern‐based PUFs, which become susceptible to replication once their structural features are exposed.

Note that the key requirement for strong optical PUFs is the generation of multidimensional physical complexity within a precisely controlled fabrication framework. This necessitates the incorporation of emitters capable of inducing photophysical variations, typically manifested as changes in emission color and modulation of missive lifetimes, arising from phase transitions [14] and interfacial interactions within heterogeneous systems [15, 16]. Perovskites are well‐known compound semiconductors that exhibit strong sensitivity to subtle environmental stimuli, enabling dynamic modulation of their physical properties through changes in crystal structure and packing [17]. Accordingly, perovskite emitters realize tunable emission characteristics via reconfiguration of electronic states associated with transition metal coordination environments [18]. Among these materials, cesium manganese bromide (Cs_x_MnBr_y_, *x* = 1 or 3 and *y* = 3 or 5) systems [19, 20, 21, 22], which are lead‐free and solution‐processible, are particularly attractive for optical PUF applications due to their low toxicity and versatile photophysical tunability. In these systems, Mn^2+^ ions, possessing a 3d^5^ electronic configuration, play a central role in governing the emission behavior through crystal field‐dependent energy splitting and spin‐forbidden d‐d transitions, enabling broad tunability in emission wavelength, lifetime, and quantum efficiency [19, 22].

In this study, we demonstrate a multiband optical PUF based on a mixed‐phase perovskite system integrated onto a Korean traditional cellulose membrane (Hanji) via an eco‐friendly solution process. Cs_x_MnBr_y_ species are selectively transformed into two distinct phases of Cs_3_MnBr_5_ and CsMnBr_3_, depending on local humidity conditions governed by annealing temperature during solvent evaporation. Under humid conditions, the CsMnBr_3_ phase dominates and exhibits red emission, whereas under low‐humidity conditions, the Cs_3_MnBr_5_ phase prevails, emitting green phosphorescence with high quantum yield. For PUF operation, the phase‐dependent emission characteristics of the mixed‐phase perovskites enable the extraction of structural randomness from the underlying cellulose fiber network through optical emission imaging. Notably, both phases exhibit wavelength‐dependent optical responses, allowing simultaneous access to spectral and spatial randomness. By exploiting multiple excitation wavelengths, a strong PUF framework is realized, generating distinct random patterns from a single device and an exponentially large space of CRPs. Furthermore, authentication and image encryption/decryption are demonstrated to validate the practical applicability of multiband optical PUFs. This work establishes a photophysical PUF platform based on mixed‐phase perovskite integrated with polymeric substrates, providing a robust approach toward hardware security systems.

## 2 Results and Discussion

Figure 1a schematically illustrates the key components of the multiband optical PUFs, consisting of Cs_x_MnBr_y_ species as the emitting layer and cellulose membranes as both the substrates and the source of spatial randomness. To achieve simultaneous spectral and spatial randomness, each component in the PUF system is deliberately engineered to contribute distinct forms of physical entropy. This architecture enables strong PUF operation with an expanded challenge‐response space.

**FIGURE 1:**
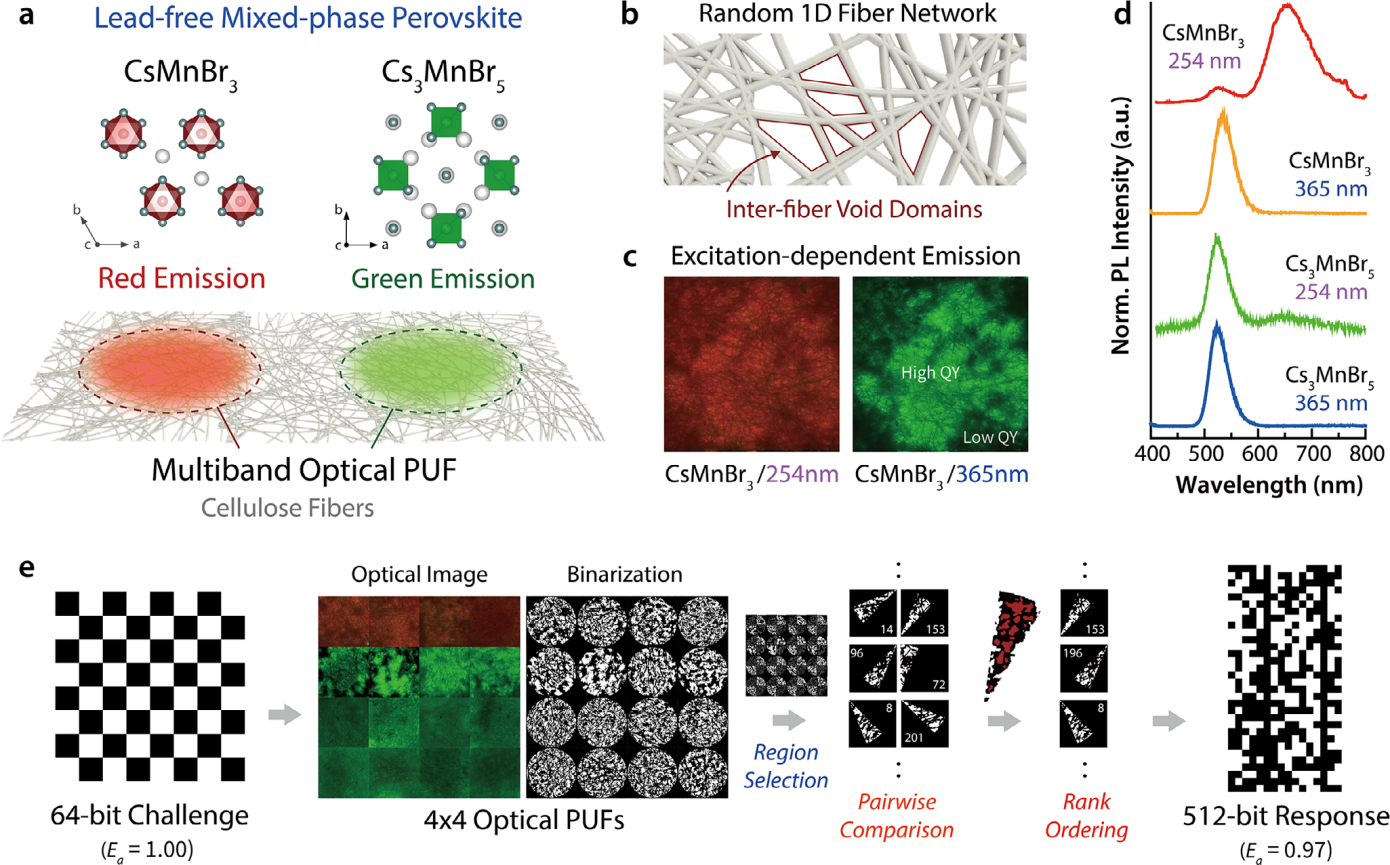
Multiband optical physically unclonable functions (PUFs) based on lead‐free mixed‐phase perovskites on cellulose fibers. (a) Schematic illustration of multiband optical PUFs formed by CsMnBr_3_ and Cs_3_MnBr_5_ phases crystallized on a cellulose membrane. (b–d) Structural and optical characteristics of the resulting heterostructures. (b) Randomly oriented one‐dimensional cellulose fiber networks and the corresponding inter‐fiber void domains. (c) Optical images and (d) photoluminescence (PL) spectra of excitation‐wavelength‐dependent multilevel emission and contrast variation arising from the coexistence of CsMnBr_3_ and Cs_3_MnBr_5_. (e) Schematic illustration of challenge (C_n_)–response (R_n_) pair generation and bit extraction.

The Cs_x_MnBr_y_ films are synthesized via an eco‐friendly solution process [21]. Briefly, CsBr and MnBr_2_ powders with a 3:1 molar ratio are dissolved in deionized water at room temperature. Then, the precursor solution is drop‐cast onto cellulose membranes, followed by mild annealing to remove the solvent. During this process, the Cs_x_MnBr_y_ system spontaneously forms two distinct phases, CsMnBr_3_ and Cs_3_MnBr_5_. They are driven by the coordination instability of Mn^2+^ ion in Mn‐based perovskite derivatives [14, 19]. In both phases, Cs^+^ ions are spatially isolated from the Mn‐Br sublattices and do not contribute to the emission characteristics [19, 23]. CsMnBr_3_ consists of octahedral [MnBr_6_]^4−^ units, whereas Cs_3_MnBr_5_ is composed of [MnBr_4_]^2−^ units, as illustrated in Figure 1a inset [14, 20, 23, 24].

The optical properties of Mn halide perovskites, including Cs_x_MnBr_y_, are primarily governed by the 3d^5^ electronic configuration of Mn^2+^ ions, which give rise to characteristics of spin‐forbidden d–d transition (^4^T_1_ → ^6^A_1_). In particular, the coordination number of Mn^2+^ is a key parameter influencing emission behavior. As the covalency of the Mn─Br framework increases, the Mn d orbitals become more delocalized toward Br^−^ ions, leading to reduced electron‐electron repulsion within the d orbital. Consequently, the Racah B, which quantifies the inter‐electronic repulsion in d orbitals, decreases, resulting in the stabilization of the excited states and a redshift in the emission wavelengths [19, 23, 24].

Through the solution process, CsMnBr_3_ layers formed under both ambient drying and annealing at temperatures below 150°C. Owing to the high coordination number of Mn^2+^, CsMnBr_3_ emits red luminescence under 254 nm excitation. In contrast, Cs_3_MnBr_5_ films are preferentially formed upon annealing at 150°C under the proposed fabrication conditions. These films exhibit bright green emission with a quantum yield (QY) of 66%. Despite the low process temperature, this QY is comparable to that of films synthesized via chemical vapor deposition at 900°C [20]. Notably, the relative proportion of CsMnBr_3_ and Cs_3_MnBr_5_ in the mixed‐phase films relies on the annealing temperatures during solvent evaporation.

Figure 1b,c presents the spatial and spectral randomness of the Cs_x_MnBr_y_/cellulose heterostructures for multiband optical PUFs. Spatial randomness originates from the one‐dimensional fibrous network of cellulose, where randomly interconnected fibers form irregular polygonal void domains that serve as primary physical disorder features. Additional randomness arises from the multilevel emission of mixed‐phase perovskite films on the cellulose substrate. Depending on the excitation wavelength, even a single device produces distinct emission patterns, as shown in Figure 1c,d. Especially, CsMnBr_3_‐dominant heterostructures exhibit red emission under 254 nm excitation but show green emission under 365 nm excitation. On the contrary, Cs_3_MnBr_5_‐dominant heterostructures consistently emit green light with variations in QYs under both excitation conditions. These variations in emission color and QY introduce additional degrees of photophysical variability, enabling higher‐dimensional randomness and expanding the entropy of the resulting PUF system. The heterostructure of the multiband‐optical PUFs enables the spatial randomness originating from both the Hanji substrate and Cs_x_MnBr_y_ layers to be combined with the distinct emission properties of each phase, thereby yielding different photoluminescence images from the same PUF device, as shown in Figure S1.

Figure 1e illustrates the CRP generation and bit‐stream extraction for the multiband optical PUFs. Challenges (*C*_n_) are arbitrarily created as 64‐bit binary sequences, in which “0” and “1” are uniformly distributed, corresponding to a total challenge space of 1.83 × 10^18^ (equivalent to $\begin{array}{c} 64\\ 32 \end{array}$). The randomness of each bit stream is quantified using the Shannon entropy (*E*_α_) determined by the relative proportions of ‘0’ and ‘1’bits [25]. An ideal binary bit stream with equal probabilities of the two binary states yields the *E*_α_ value of 1.00. Accordingly, all the challenges exhibit the ideal *E*_α_ value. Each 64‐bit *C*_n_ is applied to a 4 × 4 Cs_x_MnBr_y_/cellulose PUF array. An individual PUF is angularly partitioned into 16 segments with an interval of 22.5^○^, indexed by *n* (*n* = 0, 1, …, 15). The full 4 × 4 array is combined to form a total of 256 segments (*n* = 0, 1, …, 255), where each segment is assigned to a value corresponding to the number of inter‐fiber void domains within that region. Upon applying a given *C*_n_, segments corresponding to “1” bits are selectively extracted and sorted. The ordered segment indices are then concatenated to form a decimal sequence, which is subsequently converted into a binary bit‐stream. The resulting responses (*R*_n_) are obtained as 512‐bit sequences with *E*_α_ of 0.97, close to the ideal value of 1.00. Further details of the preprocess and bit extraction process are provided in the Experimental section, Figure S2, and Supplementary Note S1.

The mixed‐phase perovskites are fabricated, and the effects of processing conditions on phase formation and optical properties are explored. Figure 2a shows the emission images of Cs_x_MnBr_y_ powders annealed at 80 and 150°C under 254 and 365 nm excitation. For comparison, CsBr and MnBr_2_ precursors are mixed at different molar ratios and subjected to annealing at these temperatures. Samples annealed at 80°C exhibit emission color variations, showing red emission under 254 nm excitation and green emission under 365 nm excitation. In contrast, samples annealed at 150°C display consistently bright green emission regardless of the excitation wavelength.

**FIGURE 2:**
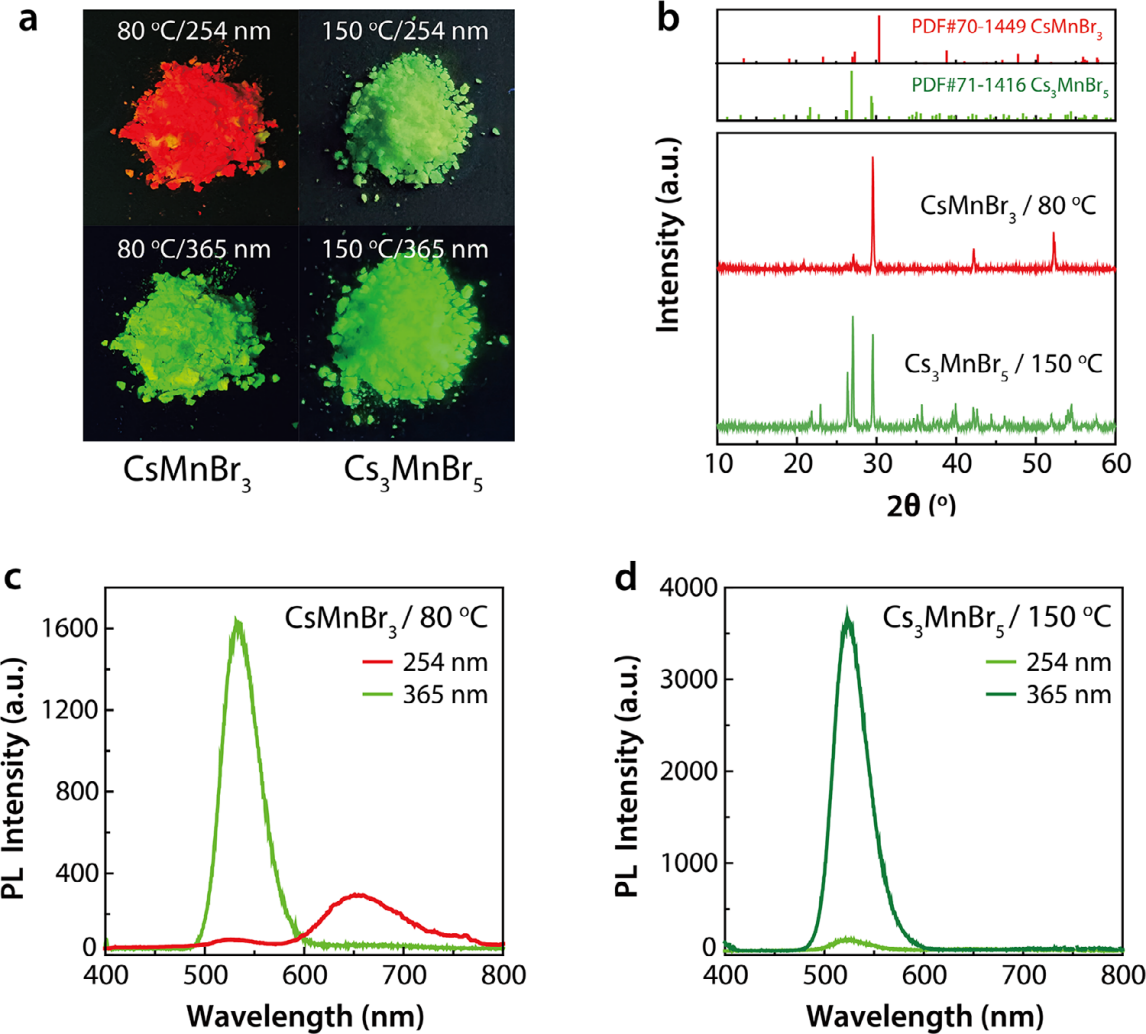
Crystal structure and optical properties of CsMnBr_3_ and Cs_3_MnBr_5_ powders formed under different annealing temperatures. (a) Photographs of the powders under UV excitation wavelengths (λ_*ex*_ = 254 and 365 nm) at annealing temperatures of 80°C and 150°C. (b) X‐ray diffraction patterns of CsMnBr_3_ and Cs_3_MnBr_5_ powders. (c,d) PL spectra of (c) CsMnBr_3_ and (d) Cs_3_MnBr_5_ under different excitation wavelengths (λ_*ex*_ = 254 and 365 nm).

As shown in Figure 2b, the X‐ray diffraction (XRD) patterns of the red‐emitting powders show diffraction peaks at 27.0 and 30.4^○^, corresponding to the (200) and (201) planes of CsMnBr_3_ with a P6_3_/mmc space group [26]. The green‐emitting powders show prominent peaks at 26.3, 27.0, and 29.5^○^, which are assigned to the (114), (213), and (310) planes of Cs_3_MnBr_5_ with a I4/mcm space group [26]. Notably, additional minor diffraction peaks are observed in both cases, indicating that the powders consist of mixed‐phase perovskites with dominant CsMnBr_3_ or Cs_3_MnBr_5_ phases depending on the annealing condition.

Figure 2c,d presents the photoluminescence (PL) spectra of the mixed‐phase powders under different excitation wavelengths. CsMnBr_3_‐dominant powders exhibit a green emission centered at 532.9 nm with a full width at half maximum (FWHM) of 43.3 under 365 nm excitation. Under 254 nm excitation, a pronounced red emission peak emerges at 654 nm with a broader FWHM of 94.4 nm accompanied by a weaker emission feature near 522 nm. The overall PL intensity decreases one quarter. The red emission is attributed to the stabilization of the excite states associated with the octahedral of [MnBr_6_]^4−^ sublattices which lowers the emission energy due to enhanced crystal field interactions [19, 24]. However, the green emission observed under 365 nm excitation cannot be solely explained by the intrinsic optical characteristics of CsMnBr_3_. Instead, it is likely influenced by the presence of secondary phases or defect‐related states within the mixed‐phase system. Further discussion on this matter will be provided in the following section.

Cs_3_MnBr_5_‐dominant powders, on the other hand, exhibit consistent green emission, with PL peaks at 523.3 nm and a FHWM of 39.2 nm under both excitation wavelengths. However, the PL intensity decreases by approximately an order of magnitude under 254 nm excitation. This green emission arises from the typical optical characteristic of Cs_3_MnBr_5_ [20, 21]. The relatively narrow FHWM suggests reduced nonradiative recombination with the increased Mn‐Mn separation in the structural isolated tetragonal [MnBr_4_]^2−^ sublattices [22]. The reduced PL intensity under 254 nm excitation can be understood in terms of the limited penetration depth of higher‐energy photons, together with other optical and material‐derived factors, such as surface and non‐radiative recombination under ambient conditions [27]. Shorter excitation wavelengths generally correspond to higher absorption coefficients, leading to carrier generation confined near the surface region. As a result, the effective excitation volume is reduced to lower overall PL intensity [27]. These findings highlight the spectral complexity of the mixed‐phase system, which is essential for enhancing the entropy of the PUF platform.

To ensure scalability and compatibility with large‐area fabrication, a solution‐based drop‐casting method is employed to fabricate multiband optical PUFs. For PUF applications, in addition to the multiband optical response of mixed‐phase perovskites, we utilize Hanji (a Korean traditional cellulose membrane) as a flexible substrate, leveraging its structural randomness to introduce spatial complexity. A precursor solution of CsBr and MnBr_2_ with a fixed molar ratio of 3:1 is prepared and stirred prior to drop‐casting onto the cellulose substrate. Note that this process is environmentally benign, as diluted water is used as the solvent. Although the precursor solution favors the formation of Cs_3_MnBr_5_, interactions with the cellulose matrix during the annealing process lead to the coexistence of both CsMnBr_3_ and Cs_3_MnBr_5_ phases.

Hanji is composed of a network of one‐dimensional cellulose nanofiber [28]. Its confined geometry and high surface‐to‐volume ratio induce capillary‐driven enrichment of the precursor solution, promoting localized and heterogeneous nucleation of perovskite nanocrystals at multiple sites rather than uniform thin‐film growth [29, 30]. It is advantageous to realize highly disordered emission patterns, thereby enhancing the entropy of the system for PUF applications.

Figure 3a illustrates the crystallization behavior of Cs_x_MnBr_y_ on cellulose under different annealing conditions. Upon drop‐casting, the precursor solution spreads along the one‐dimensional cellulose fibers while abundant hydroxyl groups (‐OH) on the fiber surfaces facilitate hydrogen bonding with the solvent [31]. This interaction enhances solution wetting and promotes diffusion of the precursor, enabling localized crystallization. The subsequent nanocrystals are physically anchored and interlocked within the entangled fiber structures, providing mechanical robustness and flexibility under tensile and compressive deformation. In addition, cellulose plays an important role in regulating the local moisture environment during crystallization. Under low‐temperature annealing or ambient drying conditions, water molecules bonded to surficial hydroxyl groups remain during nucleation and crystal growth. Such conditions favor higher coordination states of Mn^2+^ ions [14, 32], increasing the relative fraction of CsMnBr_3_. In contrast, higher temperature annealing accelerates solvent evaporation, leading to a more dehydrated local environment within the cellulose matrix. These conditions favor the formation of Cs_3_MnBr_5_ nanocrystals. Elevated thermal energy also improves crystal packing and alignment while reducing defects‐related nonradiative transition associated with residual precursors.

**FIGURE 3:**
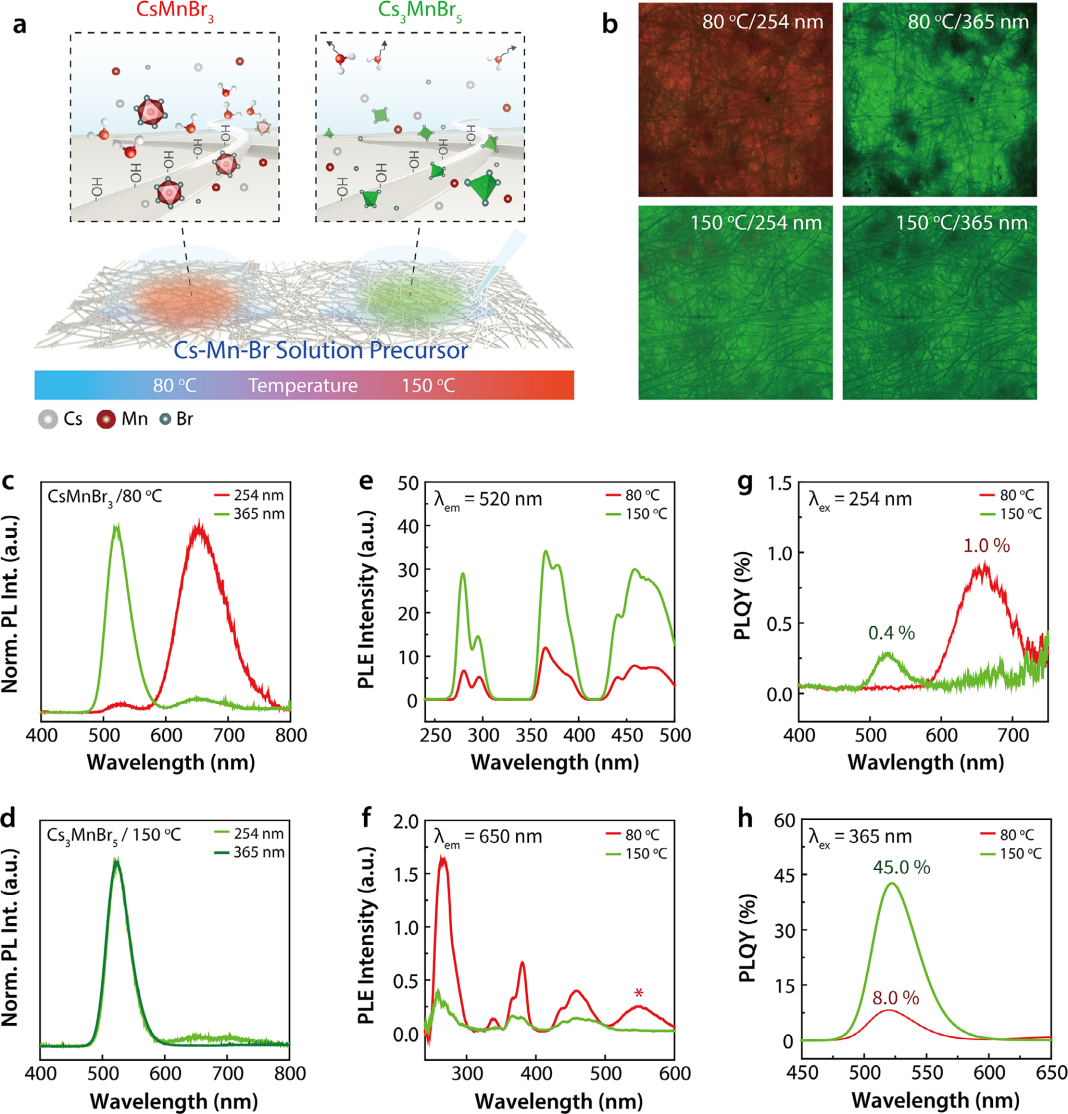
Mixed‐phase CsMnBr_3_ and Cs_3_MnBr_5_ on cellulose fibers. (a) Schematic illustration of the crystallization mechanism of mixed‐phase perovskites at the cellulose interface upon drop‐casting of Cs─Mn─Br precursor solutions under different annealing temperatures. (b) Emission images of Cs_3_MnBr_5_‐dominant and CsMnBr_5_‐dominant films under 254 and 365 nm excitation. (c, d) PL spectra of (c) Cs_3_MnBr_5_‐dominant and (d) CsMnBr_5_‐dominant films under 254 and 365 nm excitation. (e, f) PLE spectra measured at the emission wavelengths of (e) 520 and (f) 650 nm. (g, h) PLQY spectra under (g) 254 and (h) 365 nm excitation.

The interplay between the cellulose substrate and annealing conditions contributes to tailoring the phase composition of Cs_x_MnBr_y_. As supported by the XRD patterns (Figure S3), the resulting films consist of mixed phases with dominant compositions depending on processing conditions. Importantly, the presence of minor or defective Cs_x_MnBr_y_ phases is considered beneficial in this work, as they can introduce additional emissive pathways through selective excitation and interphase energy transfer.

Figure 3b shows optical images of emissive mixed‐phase perovskite films on cellulose under different processing conditions. Consistent with the powder samples, CsMnBr_3_‐dominant films exhibit excitation‐dependent emission characteristics (Figure 3c), whereas Cs_3_MnBr_5_‐dominant films predominantly show green emission with only a weak contribution from defect‐related states under 254 nm excitation (Figure 3d). To further elucidate these emission dynamics, photoluminescence excitation (PLE) spectroscopy is performed for both films by monitoring emission at 520 and 650 nm, corresponding to Cs_3_MnBr_5_ and CsMnBr_3_. As shown in Figure 3e, both films access similar PLE features when observed at 520 nm. The PLE peaks at 295.0, 365.8, 378.2, and 458.2 nm are associated with electronic transitions from the ground state (^6^A_1_) to excited states, such as ^4^A_2_(F), ^4^T_1_(P), ^4^E(D), ^4^A_1_(G), and ^4^T_1_(G) [19, 24, 32]. It indicates that despite variations in excitation wavelength, both systems predominantly have green emission associated with Cs_3_MnBr_5_, although their relative contributions may vary depending on relative phase compositions.

Unlikely, the PLE features are considerably altered when monitoring emission at 650 nm, corresponding to the CsMnBr_3_‐related emission [19] (see Figure 3f). For the films annealed at 150°C, the PLE response at 650 nm largely follows excitation pathways of Cs_3_MnBr_5_, with minor contributions from secondary phases. In contrast, the CsMnBr_3_‐dominant films formed at 80°C have pronounced PLE peaks associated with CsMnBr_3_, including excitation via ^4^A_2_, ^4^E, ^4^T_2_, and ^4^T_1_ states from the ^6^A_1_ ground state [24]. Notably, the ^4^T_1_ → ^6^A_1_ transition observed around 550 nm (marked in Figure 3f) represents a distinct characteristics of CsMnBr_3_, which spectrally overlaps with the emission of Cs_3_MnBr_5_ domains. This spectral overlap suggests the possibility of reabsorption or interphase energy transfer processes between neighboring phases in close proximity.

Time‐resolved PL (TRPL) measurements are conducted to examine whether spectral overlap between the emission of Cs_3_MnBr_5_ and the excited states of CsMnBr_3_ induces quenching of Cs_3_MnBr_5_ domains within CsMnBr_3_‐dominant films via interphase energy transfers (Figure S4). Because both films consist of mixed phases, the measured lifetimes reflect contributions from multiple emissive species. In turn, the emissions at 520 and 650 nm are primarily attributed to Cs_3_MnBr_5_ and CsMnBr_3_, respectively.

In Cs_3_MnBr_5_‐dominant films, the emission lifetime at 650 nm (681 µs) is significantly longer than that at 520 nm (324 µs), indicating less efficient recombination for the red‐shifted emission. This observation is consistent with the weak PLE response at 650 nm. For the 80°C‐annealed films, the emission lifetime at 650 nm is approximately 112 µs, in good agreement with previously reported CsMnBr_3_ lifetimes, which are sensitive to Mn‐Mn interactions and local coordination environments [33]. At 520 nm, a shorter lifetime (196 µs) is observed compared to that of Cs_3_MnBr_5_‐dominant films. This reduction in the Cs_3_MnBr_5_ emissive decay implies that both reabsorption and interphase energy transfer processes from Cs_3_MnBr_5_ to CsMnBr_3_ domains are operative. These processes likely contribute to the multicolor emission behavior in the 80°C‐annealed films.

Figure 3g,h further support these observations. Under 365 nm excitation, both films exhibit relatively high QYs up to 45% and 8% for the 150°C and 80°C‐annealed films, respectively. Under 254 nm excitation, the Cs_3_MnBr_5_‐dominant films show a significantly reduced QY of 0.4% at 520 nm, indicating inefficient excitation of the green emission under this condition. The 80°C‐annealed films show a broad QY distribution centered around 650 nm, with a relatively higher value of 1.0%, promoting their red emission. These results highlight the excitation‐dependent multilevel emission behavior of mixed‐phase perovskite films on cellulose. Such spectral diversity provides an additional degree of freedom for generating CRPs within a single PUF device, thereby increasing the dimensionality and entropy of the system for hardware security applications.

To demonstrate the applicability of mixed‐phase Cs_x_MnBr_y_ on cellulose substrates as a PUF platform, the spatial randomness of inter‐fiber void domains is exploited as a key physical parameter. To ensure sufficient complexity and irreproducibility of these patterns, an image‐processing workflow is implemented as illustrated in Figure S5. Initially, emission images of Cs_x_MnBr_y_/cellulose heterostructures are acquired using a charge‐coupled device (CCD) camera equipped with multiple excitation lasers. The excitation‐dependent optical characteristics of the Cs_x_MnBr_y_ layers selectively reveal different regions of the underlying cellulose nanofiber network. Even when the emission wavelength is similar, differences in the PLQY produce subtle local intensity variations that are readily resolved by the CCD imaging system. These images are subsequently converted into binary images through preprocessing, including grayscale conversion and adaptive local threshold functions. In adaptive thresholding, the pixel‐wise threshold *T*(x,y) is determined from the statistical properties of a neighboring window *W* surrounding each pixel (*x*, *y*). This method is particularly effective for images affected by spatial intensity variations arising from depth‐of‐focus limitations and non‐uniform illumination and has been widely adopted in image‐ or pattern‐based PUFs [34, 35].

However, in our system, the cellulose fiber network with localized emission introduces additional complexity, as the signal is strongly influenced by variations in fiber thickness, depth of focus, and illumination non‐uniformity. These factors make it challenging to reliably distinguish foreground from background using conventional global threshold approaches. Therefore, adaptive local thresholding is implemented to compensate for spatial intensity inhomogeneities and to enable robust extraction of binary patterns.

Figure 4a presents the binary images of multiband optical PUFs based on Cs_x_MnBr_y_/cellulose heterostructures, derived from the emission images shown in Figure 3b. Notably, the same sample yields distinct binary patterns under different excitation conditions, while patterns obtained from different PUF devices are inherently unique and irreproducible. As aforementioned (Figure 1e), these binary patterns are further partitioned into 16 segments and the inter‐fiber void domains within each segment are quantified and indexed by segment number *n*. To evaluate randomness, the distributions of inter‐fiber object counts across segments *n* are analyzed (see Figure 3b). All regions exhibit relatively uniform count distributions across multilevel PUFs, indicating the absence of spatial bias and confirming that each binary pattern is statistically unique and distinctive.

**FIGURE 4:**
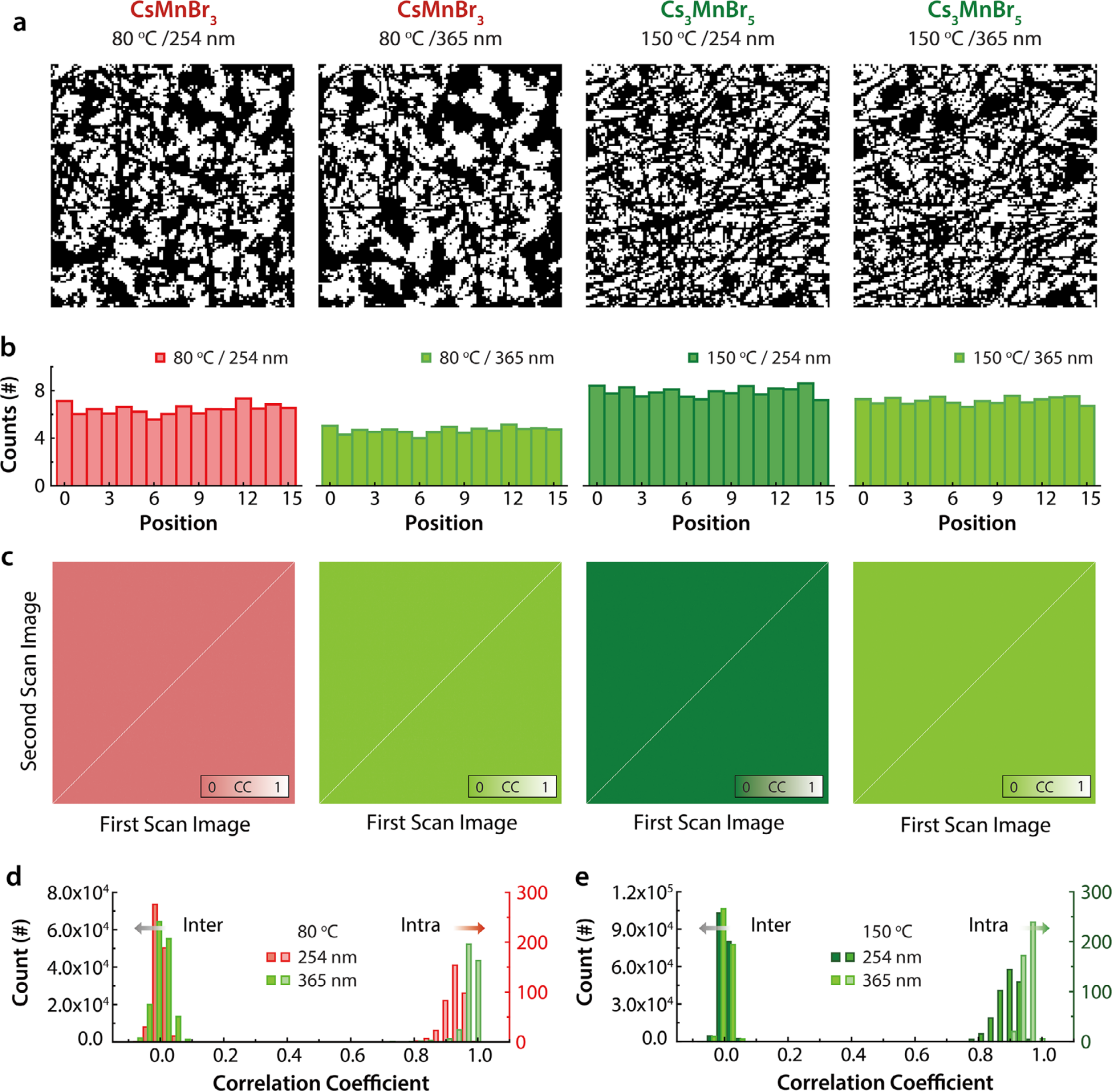
Physical randomness evaluation of multiband optical PUFs based on CsMnBr_3_ and Cs_3_MnBr_5_ on cellulose membranes. (a) Binary images of multiband optical PUFs under the conditions presented in Figure 3b. White regions represent inter‐fiber void domains and black parts correspond to cellulose fiber networks. (b) Histograms of the number of valid inter‐fiber void domains at positions (0–15) within a single binary image. (c) Two‐dimensional (2D) cross‐correlation heatmaps. (d, e) Histograms of the 2D cross‐correlation coefficient distributions for (d) CsMnBr_3_‐dominant PUFs and (e) Cs_3_MnBr_5_‐dominant PUFs. The total sample sizes of binary images analyzed in (c–e) are 368, 396, 436, and 436 for the optical PUFs annealed at 80°C and 150°C and acquired under 254 and 365 nm excitation, respectively.

Moreover, spatial correlations of inter‐ and intra‐PUFs are evaluated by acquiring emission images of individual PUFs at different orientations and calculating the corresponding cross‐correlation coefficients (CC). In the two‐dimensional correlation heatmap (Figure 4c), CC values of ‘1’ and ‘0’ represent perfect correlation and no correlation between two examined PUFs, respectively. Figure 4d,e presents statistical histograms of CC values under 254 and 365 nm excitation. In intra‐PUF comparisons, the CC values are predominantly centered near 1, indicating reliability of PUF devices and the image acquisition. Inter‐PUF comparisons show CC values centered around 0, demonstrating clear distinction between different PUF devices and confirming strong randomness. These results indicate that the multiband PUFs exhibit negligible inter‐device correlation, arising from the intrinsic uniqueness and irreproducible spatial randomness of the cellulose nanofiber network, thereby highlighting their potential for robust and secure hardware‐based PUF applications. In addition, the response uniformity is evaluated for PUFs annealed at 80 and 150°C under 254 and 365 nm excitation (Figure S6). An ideal uniformity of 0.5 corresponds to equal proportions of ‘0’ and ‘1’ bits, and the mean response uniformity ranges from 0.4967 to 0.4990. These values indicate negligible bit bias in the generated responses, with no preferential generation of either binary state.

Next, we demonstrate strong PUF operation by leveraging multilevel emission enabled by the multiband excitation and emission characteristics of mixed‐phase Cs_x_MnBr_y_ emitters. Conventional pattern‐based PUFs are vulnerable to counterfeiting because their static patterns, once exposed, can be replicated. This limitation motivates the incorporation of additional random features that are dimensionally independent of the spatial patterns themselves. This approach enables strong PUF operation, in which a virtually unlimited number of CRPs can be generated from a single device. These multidimensional tuning parameters serve as independent knobs, realizing exponential CRP generation in conjugation with programmable logic schemes [36].

Figure 5a presents the strong PUF model for multiband optical PUFs based on mixed‐phase Cs_x_MnBr_y_/cellulose heterostructures. Initially, each PUF device contains multilevel encodable datasets composed of distinctive emission patterns, governed by annealing temperatures (*T*_an_)‐dependent phase composition as well as excitation wavelengths (*λ*_ex_)‐dependent emission characteristics. When a 64‐bit *C*_n_ is applied, the device generates a 512‐bit response (*R*_n,T,λ_) for a selected combination of *T*_an_ and *λ*_ex_, which subsequently defines the cryptographic key (*k*_n,T,λ_). Details of the CRP generation process are provided in Figure 1e and the Experimental Section. Figure 5b illustrates the strong PUF operation using a 4 × 4 multiband optical PUF array (PUF_150_) fabricated at 150°C. Distinct *C*_n_ sequences (n = 1, 2, …, n) are introduced to the PUF_150_, yielding clearly distinguishable *R*_n,150,λ_ (n = 1, 2, …, n, and *λ* = 254 and 365).

**FIGURE 5:**
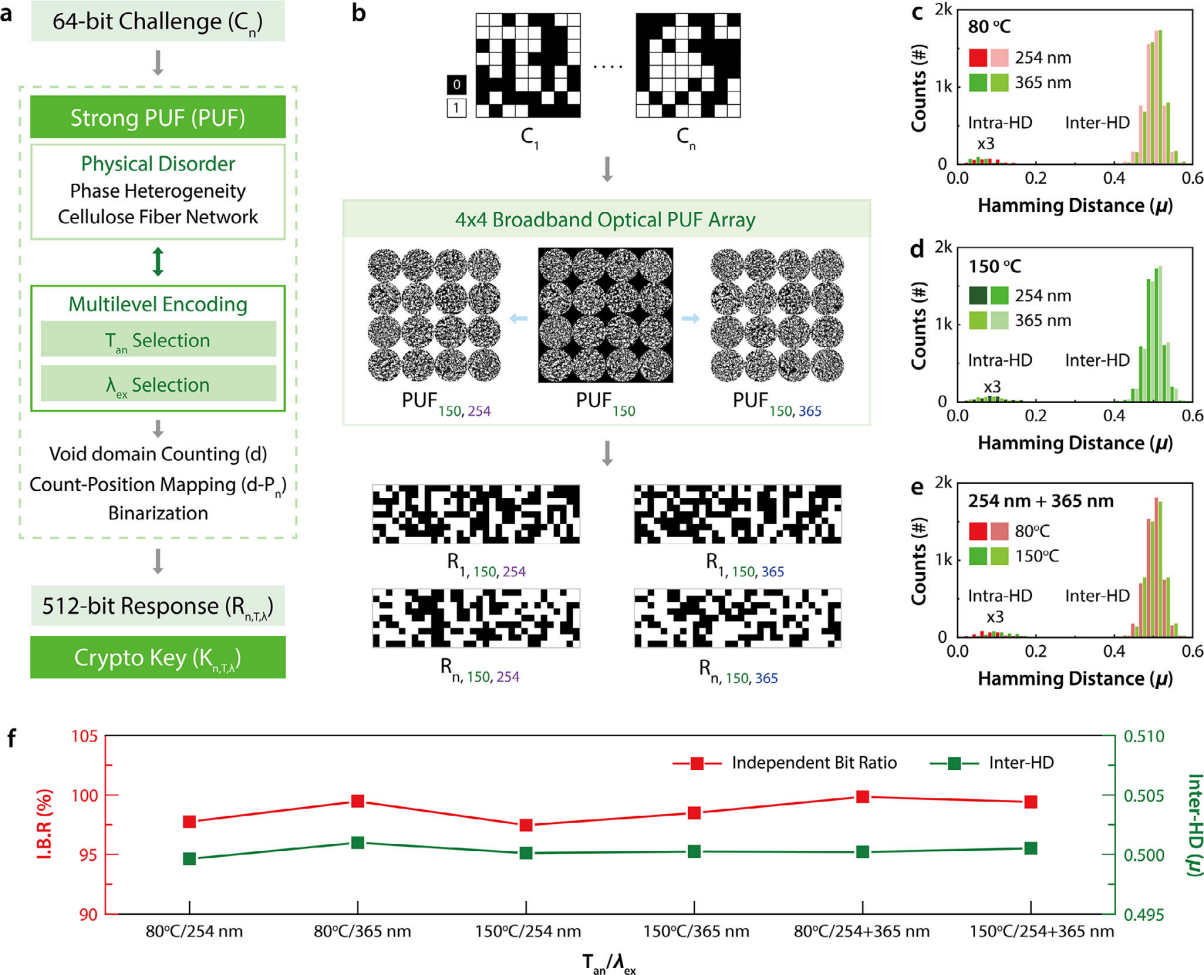
Strong PUF model based on multiband optical PUFs. (a) Schematic flowchart of the strong PUF model, utilizing annealing temperature (*T*_an_) and excitation wavelengths (λ_ex_) as multilevel encoding parameters. (b) Illustration of CRP generation under strong PUF operation. (c, d) Distributions of inter‐ and intra‐Hamming distances (HD) for multiple challenges (*C_n_*, *n* = 4) and strong PUFs (PUF*_n_*_'_, *n*' = 18) in (c) PUFs annealed at 80°C and acquired under 254 and 365 nm excitation (PUF_n,80°C, λ_), (d) PUFs annealed at 150°C and acquired under 254 and 365 nm excitation (PUF_n,150°C, λ_), and (e) PUFs formed under different annealing temperatures and excitation wavelengths (PUF_n,T, λ_). (f) Independent bit ratios (I.B.R) and inter‐HDs extracted from all PUF_n,T,λ_ cases.

To evaluate the viability and randomness of the strong PUFs for cryptographic applications, a total of 72 *R*_n,T,λ_ are obtained from 18 PUF arrays by applying four different *C*_n_ and analyzed in terms of hamming distance (HD), as shown in Figure 5c,d, and Figure S7. The HD between two PUF_n'_ devices quantifies the number of different bits between their responses. Ideally, the average inter‐HD and intra‐HD should reach 0.5 and 0, respectively, representing maximal uniqueness and high tolerance against environmental noise. The HD is calculated by $\frac{1}{l}\sum_{i\;=\;1}^{l}\left(B1_{i}⊕B2_{i}\right)$, where *B*1_*i*_ and *B*2_*i*_ denote the *i_th_* bits of two *R*_n,T,λ_, and *l* is the total bit length [12].

All PUF_n,T,λ_ configurations, including those generated from hybridized emission patterns under combined excitation conditions (254+365 nm) as shown in Figure 5e, exhibit inter‐HD values of approximately 0.5 and intra‐HD values near 0. The corresponding average (μ) and standard deviations (σ) under each condition are summarized in Table 1. Based on these HD statistics, the degree of freedom (DoF) is extracted by μ · (1 − μ)/σ^2^ from which the encoding capacity is estimated as 2^*DoF*^ [12]. Accordingly, the encoding capacities of PUF_n,80, λ_, PUF_n,150, λ_, and PUF_n,T, 254+365_ are calculated and listed in Table 2. These results demonstrate that the multiband optical PUFs successfully operate as strong PUFs and enable an exponential increase in the number of CRPs from a limited number of PUF devices. Moreover, the system achieves independent bit ratios of up to near 100% while maintaining the inter‐HD averages of approximately 0.5, as shown in Figure 5f. In addition, the false positive and false negative rates range from 10^−14^ to 10^−27^, indicating the performance of the multiband optical PUF platforms.

**TABLE 1:** The average and standard deviations of inter‐ and intra‐HD distributions, DoF, and independent bit ratios of the *R*_n,T, λ_. All the results are extracted from the results shown in Figure 5c–f.

T_an_/ *λ*_ex_	Inter‐HD		Intra‐HD		DoF (I.B.R (%))
	µ	σ	µ	Σ	
80°C/254 nm	0.500	0.022	0.084	0.026	500.453 (97.74%)
80°C/365 nm	0.501	0.022	0.054	0.020	509.274 (99.47%)
150°C/254 nm	0.500	0.022	0.098	0.031	498.984 (98.49%)
150°C/365 nm	0.500	0.022	0.078	0.027	504.254 (98.49%)
80°C/254+365 nm	0.500	0.022	0.087	0.027	511.17 (99.84%)
150°C/254+365 nm	0.500	0.022	0.109	0.027	508.922 (99.40%)

**TABLE 2:** Encoding capacities of the strong PUFs shown in Figures 5c‐e under various conditions.

	PUF_n,80,254_	PUF_n,80,365_	PUF_n,150,254_	PUF_n,150,365_	PUF_n,80,254+365_	PUF_n,150,254+365_
T_AN_	80	80	150	150	80	150
λ_ex_	254	365	254	365	254+365	254+365
Encoding Capacity	4.48 × 10^150^	2.03 × 10^153^	1.62 × 10^150^	6.24 × 10^151^	7.56 × 10^153^	1.59 × 10^153^

It should be noted that the challenge does not introduce any additional randomness into the resulting responses in the multiband optical PUF system. Rather, the challenge serves only to select regions of interest within the 4 × 4 PUF for subsequent response extraction. As shown in Figure S8a, an identical challenge was applied to three different representative PUFs, yielding distinct 512‐bit responses with substantial randomness. These results demonstrate that the uniqueness and unclonability of the generated responses are determined exclusively by the intrinsic physical randomness of the optical PUFs, regardless of the applied challenge pattern. In contrast, different challenges are applied to a single PUF array to produce corresponding responses and assess their diffuseness. Figure S8b shows that a single PUF generates non‐repeating 512‐bit responses depending on the applied challenges, exhibiting high diffuseness. To quantify the diffuseness of responses generated from an individual PUF, the HD values between the responses are calculated, as shown in Figure S9. For each condition (Figure S9a), 100 different challenges yield 4950 response pairs, whose HD values are distributed mostly around 0.5, the ideal value for distinct keys. Moreover, the same analysis was performed on 18 different PUFs and the resulting mean diffuseness ranges from 0.4982 to 0.5001. This demonstrates that highly distinct responses can be obtained from different challenges even with only a single or a small number of PUFs.

Moreover, the National Institute of Standards and Technology (NIST) statistical test suite (NIST SP800‐22) is implemented on the multiband optical PUFs under all operating conditions to evaluate their cryptographic performance. The test includes (block) frequency, (longest) run, serial, approximate entropy, and cumulative sum analyses. The results, summarized in Tables 3, 4, 5, 6, show that all the PUF_n,T,λ_ successfully pass the NIST tests without any failures. It confirms that the system exhibits high entropy and large encoding capacity establishes, comparable to that of conventional cryptographic systems.

**TABLE 3:** NIST statistical analysis results of total 36 864‐bit binary sequences generated from multiple challenge (*C_n_*, *n* = 4) and PUF devices (PUF*_n_*_'_, *n*' = 18) under 80°C annealing and 254 nm excitation.

Statistical test	*p*‐value	Proportion (*p*‐value ≥ 0.001)	Results
Frequency	0.941144	144/144	Passed
Block Frequency	0.285349	143/144	Passed
Run	0.394873	142/144	Passed
Longest Run	0.009422	139/144	Passed
Serial	0.407091	141/144	Passed
Approximate Entropy	0.989586	143/144	Passed
Cumulative Sum	0.458035	140/144	Passed

**TABLE 4:** NIST statistical analysis results of total 36 864‐bit binary sequences generated from multiple challenge (C*_n_*, *n* = 4) and PUF devices (PUF*_n_*_'_, *n*' = 18) under 80°C annealing and 365 nm excitation.

Statistical test	*p*‐value	Proportion (*p*‐value ≥ 0.001)	Results
Frequency	0.003097	141/144	Passed
Block Frequency	0.248226	142/144	Passed
Run	0.027150	142/144	Passed
Longest Run	0.000601	143/144	Passed
Serial	0.231088	143/144	Passed
Approximate Entropy	0.120363	144/144	Passed
Cumulative Sum	0.008533	143/144	Passed

**TABLE 5:** NIST statistical analysis results of total 36 864‐bit binary sequences generated from multiple challenge (C*_n_*, *n* = 4) and PUF devices (PUF*_n_*_'_, *n*' = 18) under 150°C annealing and 254 nm excitation.

Statistical test	*p*‐value	Proportion (*p*‐value ≥ 0.001)	Results
Frequency	0.419522	140/144	Passed
Block frequency	0.553854	144/144	Passed
Run	0.831880	143/144	Passed
Longest run	0.525771	142/144	Passed
Serial	0.192263	144/144	Passed
Approximate entropy	0.553854	143/144	Passed
Cumulative sum	0.697600	144/144	Passed

**TABLE 6:** NIST statistical analysis results of total 36 864‐bit binary sequences generated from multiple challenge (C*_n_*, *n* = 4) and PUF devices (PUF*_n_*_'_, *n*' = 18) under 150°C annealing and 365 nm excitation.

Statistical test	*p*‐value	Proportion (*p*‐value ≥ 0.001)	Results
Frequency	0.009422	142/144	Passed
Block frequency	0.525771	144/144	Passed
Run	0.625552	143/144	Passed
Longest run	0.106491	141/144	Passed
Serial	0.625552	142/144	Passed
Approximate entropy	0.152806	142/144	Passed
Cumulative sum	0.683283	141/144	Passed

Furthermore, the robustness of the multiband optical PUF system against machine‐learning (ML)‐based modeling attacks was further validated to assess its potential for practical security applications in the era of artificial intelligence and ML. Among various ML‐based modeling attack methods, a support vector machine (SVM) [37, 38, 39] was implemented because it is widely utilized to evaluate the learnability of CRPs through supervised learning and response prediction. For comparison, an arbiter PUF [37], a representative strong electrical PUF architecture, was generated using a simulation package and evaluated under the same SVM‐based modeling attack protocol.

As presented in Figure S10, the SVM model readily achieves a prediction accuracy exceeding 90% only after training with approximately 1000 CRP data sets for the arbiter PUF system. This high accuracy indicates that the arbiter PUFs are substantially vulnerable to modeling attacks, despite providing a large number of CRPs as a strong PUF system. In contrast, the multiband optical PUFs prevent the SVM modeling from accurately predicting responses even after training with 100 000 CRP datasets with the prediction accuracy consistently saturating at approximately 55%. Considering that the ideal prediction accuracy for a secure PUF under modeling attacks is 50%, these results demonstrate the superior resistance of the multiband optical PUF platform to ML‐based modeling attacks by leveraging its intrinsic nonlinear physical randomness across all optical conditions.

Practical cryptography applications are demonstrated using multiband optical PUFs operating in the strong PUF model. Figure 6a shows an authentication process for currency verification, in which two PUF devices are physically integrated onto a 50 000 Korean won banknote. The banknote already incorporates hidden patterns based on UV‐reactive security inks as an anti‐counterfeiting feature. In addition to these patterns, two multiband optical PUFs are incorporated onto the banknote, remaining nearly invisible under ambient light. Upon UV light illumination, however, they emit distinct multilevel luminescence depending on the excitation wavelength, in conjugation with the underlying security patterns. They generate spatially disordered emission patterns and produce corresponding CRPs through bidirectional strong PUF operation, as shown in Figure 6a inset.

**FIGURE 6:**
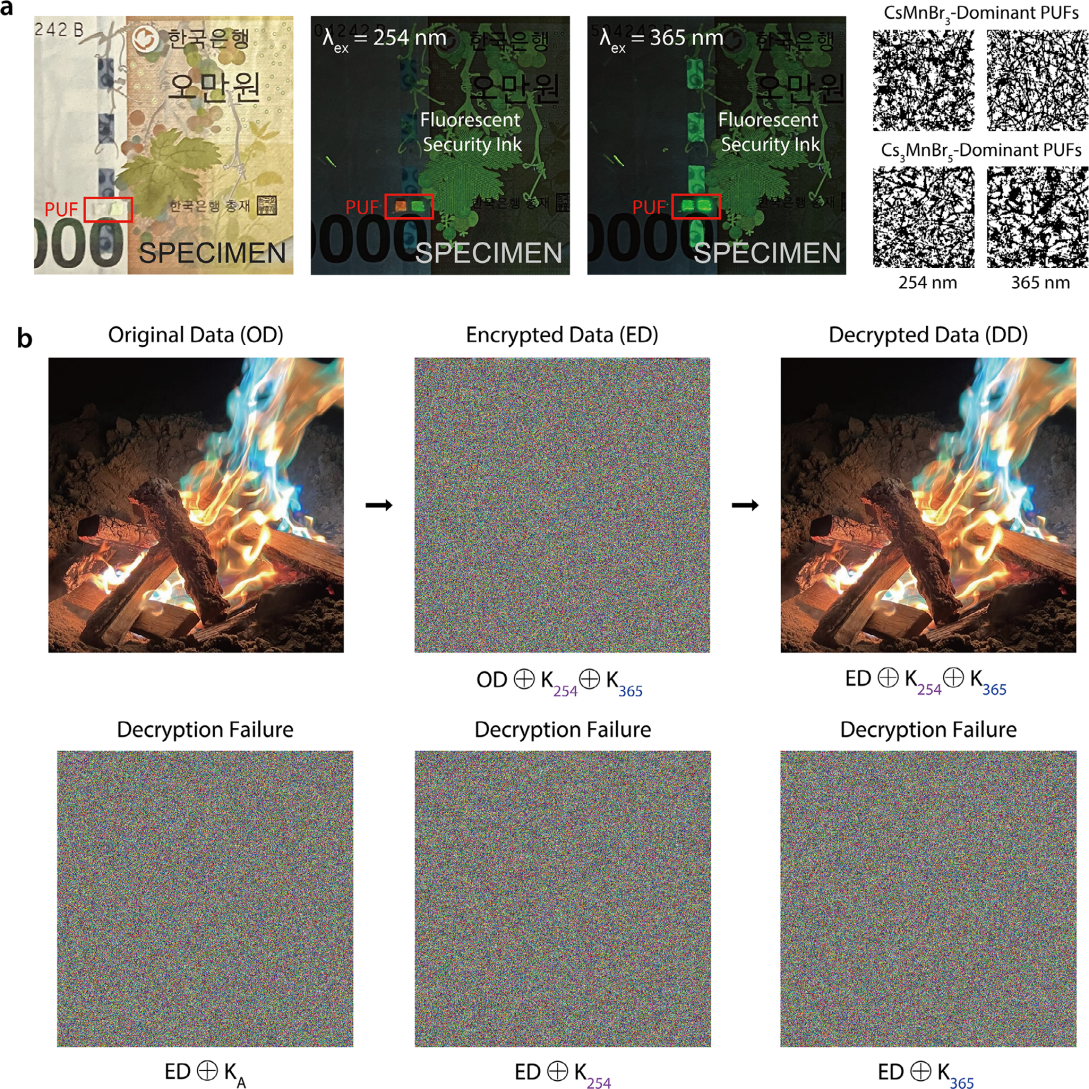
Practical applications of multiband optical PUFs. (a) Authentication process for currency verification. Two multiband optical PUFs were integrated onto a 50 000 Korean won banknote and authenticated under 254 and 365 nm excitation to verify authenticity against counterfeiting. (b) Image data encryption and decryption process. A campfire image, used as the original data (OD), is encrypted by two cryptographic keys (K_254_ and K_365_) generated from a multiband optical PUF. The encrypted data (ED) is successfully decoded with the correct key, recovering the original data. In contrast, decryption with an incorrect key produces erroneously decrypted data.

Next, we performed image data encryption and decryption using multiple cryptographic keys generated from a single PUF device under different *C*_n_, as shown in Figure 6b. Two pre‐generated keys (*k*_254_ and *k*_365_) are sequentially applied to encrypt the original data (OD) into encrypted data (ED) comprised of randomized bit streams. The encryption effectively obscures all intrinsic features of the original image, rendering it unrecognizable. For decryption the same keys must be applied in the identical order during encryption. This process reconstructs ED into the decrypted data (DD), which fully recovers the original image without any distortion. These results indicate that the ED retains the complete information content of OD, while correct keys paring and ordering are essential for recovery. In contrast, improper key usages, such as a random key or only one of the required keys, fails to reconstruct the original image, confirming the robustness and security of the encryption scheme.

Lastly, the stability evaluation of the multiband optical PUF was carried out under accelerated aging conditions to assess its potential for real‐world applications. The evaluation focused primarily on the CsMnBr_3_‐dominant devices, as the CsMnBr_3_ phase is known to be more susceptible to environmental degradation than other Cs_x_MnBr_y_ phases. Figure S11a,b presents the optical and corresponding binary images of a representative PUF after exposure to 80% RH at 80°C for up to 1 h. Throughout the aging test, the device retained both the luminescent regions of the Cs_x_MnBr_y_ layers and the underlying cellulose nanofiber networks. To systematically evaluate the reliability of the extracted binary images, cross‐correlation analyses were conducted between binary images obtained under different aging conditions, as shown in Figure S11c,d. Regardless of the aging duration, the extracted binary images exhibit CC values close to 1, indicating that the same physical patterns were consistently preserved and confirming the excellent reliability and stability of the optical PUFs even under harsh environmental conditions. As presented in Figure S6e, the stable optical characteristics enable reproducible binary code extraction and subsequent cryptographic response generation. Therefore, the mixed‐phase perovskite/cellulose optical PUFs demonstrate potential for real‐world security applications.

## 3 Conclusion

In conclusion, we demonstrate 4 × 4 multiband optical PUF arrays based on mixed‐phase Cs_x_MnBr_y_/cellulose heterostructures and their strong PUF operation. The mixed‐phase perovskites emit multilevel luminescence depending on the excitation wavelength, while the annealing conditions precisely modulate the relative phase composition of CsMnBr_3_ and Cs_3_MnBr_5_ on cellulose surfaces during solvent evaporation. Spatial randomness arises from the inter‐fiber void domains of the cellulose network. The synergistic interplay of these spectral and spatial randomness features enables strong PUF functionality, generating an innumerable number of CRPs and corresponding cryptographic keys. Moreover, those keys are utilized to perform representative cryptographic applications, including currency authentication and image data encryption and decryption. Overall, these findings highlight the potential of multilevel‐based optical PUFs as a promising platform for next‐generation security systems.

## 4 Methods

### 4.1 Fabrication and Characterization of Mixed‐phase Perovskites on Cellulose Fibers

CsBr (99.999%, Sigma‐Aldrich) and MnBr_2_ (98%, Sigma‐Aldrich) were purchased and mixed in a 3:1 molar ratio in diluted water to prepare a 1.0 M precursor solution. The solution was filtered using a syringe filter with a pore size of 0.45 µm prior to use. Hanji, a traditional Korean cellulose membrane, was obtained from the Hanji Culture Foundation [40]. The prepared solution of 20 µL was drop‐cast onto the Hanji substrates cut into 500 µm × 500 µm squares, followed by annealing at 80°C and 150°C overnight.

All PL and TRPL measurements were carried out using a laser confocal microscope equipped with photomultiplier tube detectors, where the emission from samples was collected through an optical fiber. The emission spectra were simultaneously recorded using an SR‐303I‐B spectrograph (Andor). PLQY and PLE analyses were performed through a spectroscopic platform (JASCO ILF‐825) equipped with an integrating sphere. X‐ray diffraction (XRD) measurements were conducted using a SmartLab SE diffractometer (Rigaku, Japan) with Cu Kα radiation (λ = 1.5406 Å). The diffraction patterns were recorded over a 2θ range of 10–70° with a step size of 0.02° and a scanning speed of 3° min^−^^1^.

### 4.2 Preprocess and Challenge‐Response Pair Generation Process Using Multiband Optical PUFs

Under UV illumination, emission images were acquired and preprocessed to generate binary images, with all processing performed in Python. Raw images were first aligned in terms of orientation and tilt using edge detection. Based on the center point of each image, a region of interest (ROI) was initially extracted at a resolution of 2048 × 2048 pixels and subsequently resized to 128 × 128 pixels. The ROI was converted to a grayscale image, followed by local adaptive thresholding to produce a binary pattern. The local threshold for each pixel was determined from the intensity distribution of its surrounding pixels. In the resulting binary images, white and black regions were assigned values of 0 and 1, respectively. The binary images capture cellulose fibers (black) and inter‐fiber void domains (white), and were further masked into a circle. 16 binary images obtained from Cs_x_MnBr_y_/cellulose heterostructures were combined to form a 4 × 4 PUF array. Each binary image was then divided into 16 segments indexed by *n*, resulting in a total of 256 indexed regions across the array. The number of inter‐fiber void domains within each segment was calculated and assigned as a position‐dependent parameter.

Challenges were defined as 8 × 8 arrays with µ = 0.5, yielding a challenge capacity of more than 10^18^. When a challenge was applied to the PUF array, regions corresponding to bits with a value of 1 were sorted. These regions were then paired and pairwise comparisons were performed based on the number of void domains. For each pair, the region with the higher void count was retained, forming an ordered sequence. The resulting decimal sequence was subsequently converted into a binary bit stream, yielding a 512‐bit response.

### 4.3 Randomness Evaluation and Cross‐correlation Analysis of Multiband Optical PUFs

The spatial uniqueness of PUFs was evaluated by calculating the two‐dimensional cross‐correlation coefficient between two PUF devices. In addition, the randomness and reliability of the 512‐bit responses were assessed using inter‐hamming distance (HD) and intro‐HD analyses. Based on the HD distributions, the degree of freedom (DoF) and the corresponding encoding capacity were extracted, where encoding capacity was defined as 2^DoF^. Furthermore, the independent bit ratio was evaluated to quantify the fraction of statistically usable bits, and the results were validated using the NIST statistical test suite (NIST SP 800–22)

### 4.4 Cryptographic Applications of Multiband Optical PUFs

For currency authentication, two multiband optical PUFs were attached to a Korean banknote, and their emission patterns were collected under various excitation conditions. These patterns were converted into binary images and used as PUF representations to generate responses in conjunction with applied challenges.

For image encryption and decryption, a conventional XOR‐based operation was employed. The original image data were resized to 512 × 512 × 3. Cryptographic keys were generated in advance from a pool of responses obtained from 18 PUF arrays and 683 challenges, from which two keys were selected and sequentially applied for encryption. Decryption was performed under various conditions, including the use of the correct key pair in the proper sequence, a random key, and individual application of each key.

## Author Contributions

**Seungbeom Park**: methodology, validation, writing – original draft, software, visualization. **Jiyoun Kim**: methodology, investigation, validation, writing – original draft. **Jiyoung Boo**: writing – original draft, methodology, investigation, validation, visualization. **Giho Kim**: methodology. **Jinsik Yoon**: methodology, software, validation. **Junghyun Bae**: methodology, visualization. **Sangeun Cho**: methodology. **Wook Park**: supervision, writing – review and editing, resources. **Dong Hyuk Park**: visualization, writing – review and editing, supervision, resources. **Healin Im**: conceptualization, writing – original draft, writing – review and editing, validation, supervision, resources, visualization, investigation.

## Conflicts of Interest

The authors declare no conflicts of interest.

## Supporting information




**Supporting File**: advs78505‐sup‐0001‐SuppMat.docx.

## Data Availability

The data that support the findings of this study are available from the corresponding author upon reasonable request.
