# Supplementary material for: Multiband Optical Physical Unclonable Functions Based on Mixed‐Phase Multilevel Emissive Perovskites
Source: Adv Sci (Weinh). 2026 Nov 10:e78505. Online ahead of print. doi: 10.1002/advs.78505 (PMC13749477; doi:10.1002/advs.78505)
Supplement: Supplementary file 1 [file ADVS-9999-e78505-s001.docx]

Supplementary information

**Multiband Optical Physical Unclonable Functions Based on Mixed-Phase Multilevel Emissive Perovskites**

*Seungbeom Park^1†^, Jiyoun Kim^2,3†^, Jiyoung Boo^2†^, Giho Kim^4^, Jinsik Yoon^5^, Junghyun Bae^5^, Sangeun Cho^6^, Wook Park^5,7,8^*, Dong Hyuk Park^2,3^*, and Healin Im^1^**

^1^ Department of Applied Physics, Kyung Hee University, Yongin, 17104, Republic of Korea

^2^ Department of Chemical Engineering, Inha University, Incheon, 22212, Republic of Korea

^3^ Program in Biomedical Science and Engineering, Inha University, Incheon, 22212, Republic of Korea

^4^ Department of Chemical Engineering, Kyung Hee University, Yongin 17104, Republic of Korea

^5^ Department of Electronic Engineering, College of Engineering and Information, Kyung Hee University, Yongin 17104, Republic of Korea

^6^ Division of System Semiconductor, Dongguk University, Seoul, 04620, Republic of Korea

^7^ Institute for Wearable Convergence Electronics, Department of Electronics and Information Convergence Engineering, Kyung Hee University, Yongin, 17104, Republic of Korea

^8^ School of Electronic Engineering, College of Electronic and Information, Kyung Hee University, Yongin 17104, Republic of Korea

*^†^*These authors contributed equally to this work.

*Corresponding Authors – email: [parkwook@khu.ac.kr](mailto:parkwook@khu.ac.kr), [donghyuk@inha.ac.kr](mailto:donghyuk@inha.ac.kr), [ihl0102@khu.ac.kr](mailto:ihl0102@khu.ac.kr)

**Keywords:** physical unclonable functions, multicolor emission, perovskite

**Figure S1** Schematic illustration of the multiband optical PUF system and the physical origins of its spatial and emissive randomness.

**Figure S2** Detailed CRP generation process of the multiband optical PUF system.

**Figure S3** X-ray diffraction patterns of Hanji (a traditional Korean cellulose paper), CsMnBr_3_/Cs_3_MnBr_5_ mixed-phase, Cs_3_MnBr_5_-dominant, and CsMnBr_3_-dominant perovskites formed on Hanji.

**Figure S4** Optical properties of CsMnBr_3_-dominant and Cs_3_MnBr_5_-dominant films. (a,b) Time-resolved PL decay spectra measured at emission wavelengths of (a) 520 and (b) 650 nm. (c) Schematic energy band diagram of the mixed-phase perovskite films.

**Figure S5** Image processing workflow for binary pattern extraction from raw optical images of multiband optical PUFs.

**Figure S6** Distribution of bit-level response uniformity for PUFs annealed at 80 and 150 ℃ under 254 and 365 nm excitation.

**Figure S7** HD heatmap profiles of 72 *R*_n,T,λ_ generated from 18 PUF arrays using four different *C*_n_ under the following conditions: annealed at 80 ℃ with (a) 254nm and (b) 356 nm excitation and annealed at 150 ℃ with (c) 254 nm and (d) 356 nm excitation.

**Figure S8** (a) CRP generation using an identical challenge applied to three different PUF instances, demonstrating uniqueness. The same 64-bit challenge generates distinct 512-bit responses due to the intrinsic randomness of each PUF device. (b) CRP generation using different challenges applied to a single PUF instance, demonstrating diffuseness. Different 64-bit challenges select different units within the same PUF device, resulting in distinct 512-bit responses.

**Figure S9** Diffuseness analysis of responses generated using 100 challenges in multiband optical PUFs. (a) HD distributions obtained from one PUF for each condition using 100 challenges. (b) Mean diffuseness values of 18 PUFs under the corresponding conditions.

**Figure S10** Support vector machine (SVM)-based modeling attack evaluation of multiband optical PUFs and arbiter PUFs.

**Figure S11** Stability evaluation of Cs_x_MnBr_y_/cellulose optical PUFs under accelerated aging conditions (RH 80% and 80 ℃). Optical and corresponding binary images of a representative CsMnBr_3_-dominant PUF acquired under (a) 254 and (b) 365 nm excitation after different exposure durations. Cross-correlation heatmaps between binary images obtained at different exposure times under (c) 254 and (d) 365 nm excitation. (e) Cross-correlation statistics of 14 different PUFs as a function of exposure duration.

**Supplementary Note 1**

**Challenge-response pair generation process in the multiband optical PUF system**

Figure S2 illustrates the detailed challenge-response pair (CRP) generation process of the multiband optical PUF system. First, the emission images of the 4 × 4 PUF array are processed through centroid detection, cropping, resizing, grayscale conversion, and adaptive local thresholding to obtain binarized images. Each PUF device is then divided into 16 angular units at 22.5° intervals, resulting in a total of 256 physical units across the PUF array. These 256 units are sequentially indexed from 0 to 255, with each index encoded as an 8-bit binary value. For each physical unit, the number of inter-fiber void domains is counted and stored together with its corresponding unit index and 8-bit binary representation.

A challenge is represented by an 8 × 8 binary array corresponding to a 64-bit sequence. Each challenge pixel is mapped to four units of the PUF array. A challenge pixel with a value of “1” selects the corresponding four units for response generation, whereas a pixel with a value of “0” excludes them. For a challenge with *μ* = 0.5, 32 of the 64 challenge pixels are applied to select 128 units from the total 256 units. Subsequently, the selected 128 units are randomly grouped into 64 pairs. For each pair, the stored inter-fiber void counts are compared, and the unit with the higher count is selected, resulting in 64 selected units. These selected units are further rank-ordered according to their inter-fiber void counts, from the lowest to the highest, generating an ordered sequence of 64 unit indices.

Finally, each of the 64 rank-ordered unit indices is represented by its corresponding 8-bit binary values. The resulting 64 8-bit sequences are concatenated to generate the final 512-bit response (64 × 8 = 512 bits). Therefore, the applied 64-biut challenge determines which units participate in the response generation, while the spatial random features extracted from the binary images under different excitation conditions determine the inter-fiber void counts, subsequent rank ordering, and the final 512-bit response.
